# Migraine and tension-type headache treated with stromal vascular fraction: a case series

**DOI:** 10.1186/1752-1947-8-237

**Published:** 2014-06-30

**Authors:** Ralph Bright, Matthew Bright, Pelin Bright, Shannon Hayne, Wayne D Thomas

**Affiliations:** 1 Macquarie Stem Cells, 21b Bathurst St, Liverpool, NSW 2170, Australia; 2 Cell-Innovations Pty Ltd, 21b Bathurst St, Liverpool, NSW 2170, Australia

**Keywords:** Headache, Mesenchymal stem cells, Migraine, Stromal vascular fraction, StroMed, SVF

## Abstract

**Introduction:**

Chronic migraines and tension-type headaches are debilitating diseases affecting 1.4 to 2.2% of the population with both quality of life and economic implications. To date, the pain associated with migraine and tension-type headaches has been controlled with a range of medications, with varying levels of success. In addition, the side-effect profile of these medications, as well as their potential for addiction, has been a cause for concern for both patients and physicians.

**Case presentations:**

Four women with long histories of migraine or frequent tension-type headache that meet the International Classification of Headache Disorders criteria for Chronic Migraine or Tension-type Headaches were given a systemic treatment(s) of autologous stromal vascular fraction or autologous ‘StroMed’ isolated from lipoaspirate. StroMed is stromal vascular fraction cells prepared by ultrasonic cavitation. Two of the four patients, both of whom are Arab women aged 40 and 36 years, ceased having migraines after 1 month, for a period of 12 to 18 months. The third patient, a Slavic woman aged 43 years, had a significant decrease in the frequency and severity of migraines with only seven migraines over 18 months. The fourth patient, an Asian woman aged 44 years, obtained a temporary decrease for a period of a month and was retreated 18 months later and has been free of migraines to date for 1 month. Pain medication was typically reduced from prescribed opioid analgesia to non-steroidal anti-inflammatory drugs and paracetamol.

**Conclusions:**

This case series is the first to provide evidence of the efficacy of autologous StroMed and stromal vascular fraction in the treatment of migraine and tension-type headache. The treatment of this disease by stromal vascular fraction adds a new dimension to its clinical applicability and suggests a relatively simple treatment that may help address the symptoms of the disease. Given what is known about the components of the stromal vascular fraction and how they act, the information presented in this case series may also further our knowledge of the etiology and pathophysiology of migraine and tension-type headaches. This treatment is simple, looks to be extremely effective and has been life changing for these patients.

## Introduction

The therapeutic rationale for use of stromal vascular fraction (SVF) cells and the high content of native multi-potent adipose-derived stem/stromal cells (ASCs) within the SVF has gained recent substantial clinical interest. SVF typically has been reported to contain a heterogeneous population of cells including preadipocytes, ASCs, endothelial progenitor cells (EPCs), T-regulatory cells and M2 macrophages [1]. The method of harvesting, the site and separation of these cells may result in differing proportions of these populations. Although, a large body of work has focused on cultured mesenchymal stem cells (MSCs) or ASCs with their effect on a multitude of diseases, SVF is an easily obtainable cell population which does not require culture and can be used in an autologous manner with little risk of disease transfer or the complications arising from cultured ASCs for clinical use.

Cellular populations of SVF such as ASCs have been shown to have significant immunomodulatory capabilities, capable of homing to sites of inflammation or hypoxia and promote recovery in both animal and human models [1]. Furthermore, intravenous (IV) injection of SVF has been shown to be safe without significant adverse effects [2]. SVF from adipose tissue has also been used in clinical applications with improvements demonstrated in fat transfer, osteoarthritis and Crohn’s disease.

Several methods for separation of SVF from lipoaspirate are available and include collagenase and ultrasonic cavitation; the SVF product obtained by ultrasonic cavitation has been termed “StroMed”. Although, SVF has not previously been used in the treatment of migraines in animal or human models, we noted that some patients that were treated with SVF for osteoarthritis reported a reduction in migraine frequency and severity. A small case series was performed on patients who had failed all other migraine treatments. Treatment of autologous SVF by a medical practitioner is a procedure permitted by the Therapeutic Goods Administration of Australia (Excluded Goods Order-2011). Informed written consent was obtained from each patient and the study protocol conformed to the principles of the 1975 Declaration of Helsinki.

Migraine is a common, episodic neurological disorder. It manifests as episodes of recurrent severe headaches, typically unilateral and throbbing, which may be associated with nausea, vomiting, photophobia or phonophobia. Approximately one-third of patients with migraine also experience ‘aura’ – transient neurological symptoms which are most frequently visual, but may involve speech and/or other senses [3]. Both migraine with and without aura are three times more common in females than males [4]. Tension-type headache (TTH) differs slightly in that it is defined clinically as a mild or moderate bilateral headache, with a pressing or tightening quality. These headaches can last from 30 minutes to 7 days and are associated with phonophobia and photophobia. Both migraine and TTH are significant public health problems, impacting greatly on both individuals and society. Migraine is currently treated both acutely and prophylactically utilizing: non-steroidal anti-inflammatory drugs (NSAIDs), triptans, anti-epileptics, beta-blockers, and Ca^2+^ channel blockers [4]. Unfortunately, due to the heterogeneity of these headache syndromes–particularly migraine–the current treatments vary greatly in their efficacy [3].

While it is generally agreed that migraine is a heterogeneous neurological disorder, the detail of its pathophysiology is as yet unconfirmed with inheritance thought to be as high as 50%. A migraine attack can take place over hours to days and include four overlapping phases: premonitory, aura, headache and postdrome, with the mechanisms leading to an attack largely unknown. The aura and headache phase generally overlap with a large body of indirect evidence suggesting the development of headache relies on the activation and sensitization (A/S) of neurons in the trigeminal nerve. A/S of the trigeminal nociceptors, particularly those that innervate the meninges and large cranial blood vessels, may further propel A/S of second- and third-order central trigeminovascular neurons. As these neurons project to nuclei in the brain stem and forebrain, including the ‘pain matrix’, they are considered to be the underlying cause of both migraine pain and other migrainous symptoms, including loss of appetite and sleepiness [3]. The pain matrix consists of all the parts of the brain whose activity fluctuates when an individual is experiencing pain.

The origin of the damaging stimuli by which the meningeal receptors become activated is unclear with two central hypotheses put forward: cortical spreading depression (CSD) and neurogenic inflammation. There is increasing evidence that the brief neuronal excitation followed by prolonged inhibition of activity that spreads across the cortex, referred to as CSD, is implicated in the pathophysiology of migraine [5]. The inhibition of neuronal activity appears to be correlated with localized changes in blood flow in patients, and sustained activation of the meningeal nociceptors and central trigeminovascular neurons to occur by the extracellular release and diffusion of molecules such as potassium and hydrogen ions, and glutamate in the cortex. In addition, or alternatively, CSD may also trigger neurogenic inflammation.

The neurogenic inflammation hypothesis proposes that the release of inflammatory neuropeptides such as calcitonin gene-related peptide, substance P and neurokinin A from trigeminal sensory afferents leads to vasodilatation, mast cell degranulation and ensuing release of cytokines, platelet aggregation and extravasation of plasma proteins. Of interest, most C-type and slow A-delta-type rat dural afferents are activated and sensitized by an inflammatory soup (IF) of potassium, prostaglandins, 5-hydroxtryptamine, bradykinin and histamine [6], supporting the role of inflammation in migraine pathophysiology. In addition, these dural fibers are mechanosensitive and IF enhances their mechanosensitivity–perhaps explaining why migraine headaches are throbbing, and exacerbated by actions that increase intracranial pressure [7]. Clinical support for inflammation’s role comes from the efficacy of NSAIDs.

Recent theories surrounding TTH also implicate neuronal plasticity in its pathogenesis. As reviewed in Fumal and Schoenen [8], increased nociceptive input from pericranial myofascial tissues is thought to be important for infrequent TTH and leads to sensitization of nociceptive second-order neurons in the C2 and C3 dorsal horns and trigeminal nucleus. Whereas dysfunctional nociception in the central nervous system is proposed to be predominantly related to chronic TTH, invariably these mechanisms appear to be intermingled in the pathogenesis of TTH.

Although there exists sufficient evidence to reject the original vascular theory of migraine of dilation of extracranial arteries, vascular dysfunction is still thought to be implicated in migraine. Numerous studies have indicated migraineurs have increased vascular disease, although differences in endothelial function in migraineurs remain controversial. EPCs normally act as an endogenous repair mechanism, counteracting endothelial cell injury and replacing dysfunctional epithelium to maintain endothelial integrity after damage has occurred [9]. Reduced number and function of EPCs have been reported in migraineurs, although this is debatable. The decreased numbers could indicate a role for the vascular system in migraine pathophysiology.

## Case presentations

Patients’ adipose tissue was harvested from subcutaneous locations and they were given 15 to 30mg morphine, 1 to 2mg Ativan^®^ (lorazepam), 10mg Maxolon^®^ (metoclopramide), 50 to 100mg pethidine and 4mg dexamethasone. The area was cleaned with 0.5% 6-chlorhexidine solution and an incision made to inject the tumescent fluid by a 16-gauge cannula, which is made up of 800mg lignocaine, 1mg adrenaline and 8.4g sodium bicarbonate per liter. The adipose tissue was removed by liposuction using 3mm cannula and collected in a sterile container with a washing solution. This solution was normal 0.9% saline with 8.4g sodium bicarbonate.

The lipoaspirate was digested with collagenase or by following Cell-Innovations (Australia) standard ultrasonic cavitation proprietary protocol under sterile conditions to obtain StroMed. Enzymatic digestion was performed adding 0.05% collagenase with adipose tissue over a period of 45 to 60 minutes. Samples were then centrifuged, and washed three times before being filtered through a 100um filter (Merck Millipore, MA, USA). The supernatant was removed and the pellet resuspended in phosphate buffered saline or 0.9% saline. A sample was then taken of the SVF yield and the cells counted using a hemocytometer or the Muse Cell Analyzer (Merck Millipore, MA, USA) with a Count and Viability dye (Merck Millipore, MA, USA). The total cell number was then determined.

Plasma rich in growth factors (PRGF) was prepared by centrifugation of 2 × 10mL of blood with acid-citrate-dextrose formula A (450g × 10 minutes). The plasma was removed and the platelets further concentrated by centrifugation at 2000g for 10 minutes. The platelet-poor plasma was removed and the platelets resuspended in 1.5mL platelet-rich plasma (PRP). The PRP was clotted with the addition of 150uL of calcium gluconate (10%; Regen Lab, Lausanne, Switzerland) and after 30 minutes the PRGF was removed.

Cells were administered IV by dilution in a 1L saline bag and given intravenously over 1 hour.

### Case 1

Case 1 is a 40-year-old Arab woman who is a cigarette smoker with a lifelong history of chronic migraine and TTH, satisfying the International Classification of Headache Disorders-2 (ICHD-2) criteria. She typically experienced two migraines per day with classic aura, as well as frequent episodic TTH. She was given a single IV dose of StroMed (245 million) with PRGF added. At 3 days post-treatment she noted improvement in her migraine symptoms, and had ceased medication for her migraines at 1 month post-treatment. After 3 months post-treatment, she had experienced only one TTH. At 18 months post-treatment she experiences only occasional, minor headaches that are quickly resolved and do not keep her bedridden.

### Case 2

Case 2 is a 36-year-old Arab woman who is a cigarette smoker with a long history of migraine, satisfying the ICHD-2 criteria for chronic migraine. She typically experienced more than 20 days of migraine per month, and occasionally required home visits from a doctor for parenteral analgesia. She was given a single IV dose of StroMed (41 million cells) and reported not experiencing any migraines after 1 month post-treatment. At 12 months post-treatment, she stated she had only experienced two strong headaches, which were managed with paracetamol and no migraines.

### Case 3

Case 3 is a 43-year-old Slavic woman who is a non-cigarette smoker with a long history of migraines and headaches, satisfying the ICHD-2 criteria for chronic migraine. She experienced more than 20 days per month of migraine, and was using a combination of transdermal morphine patches, oxycodone hydrochloride, and up to eight tablets a day of Panadeine Forte^®^ (co-codamol; 30mg codeine phosphate, 500mg paracetamol) to control her symptoms. She was given a single IV dose of autologous adipose tissue-derived SVF (495 million cells), digested by collagenase. After 3 days post-treatment, she noticed a reduction in her migraine symptoms and ceased using transdermal morphine; over the next 5 months she was able to control her symptoms with ibuprofen and paracetamol. After 5 months, minor headaches returned and she was re-treated at 7 months with a further IV dose of autologous adipose tissue-derived SVF (638 million cells) with PRGF. She noticed immediate improvements in her symptoms. After a period of 18 months she had only experienced seven migraines.

### Case 4

Case 4 is a 44-year-old Asian woman who is a cigarette smoker satisfying the ICHD-2 criteria for chronic migraine, with a long history of migraines and headaches. She typically had more than 20 days per month of headaches without aura. She experienced mixed tension and migraine headaches, and was taking up to six Panadeine Forte^®^ (co-codamol; 30mg codeine phosphate, 500mg paracetamol) per day for headache-associated pain. She was given a single IV dose of autologous adipose tissue-derived SVF (546 million cells) by enzymatic extraction with added PRGF. In the first month post-administration, she only experienced one headache; it lasted for half an hour and responded to paracetamol. At 1 month, she experienced a relapse of her migraine symptoms. She had a repeat treatment after 18 months and has been free of migraines for at least 1 month afterwards.

## Conclusions

This case series provides an important novel direction in which StroMed or autologous SVF may be used in treatment of a disease. Given the very heterogeneous nature of headaches and the various proposed pathogeneses, all of which may be true, for the different headaches, we can postulate that there are a number of ways in which the characteristics of MSCs and other cellular components of SVF are able to modulate the frequency, duration and severity of migraine and TTH including cell depletion, immunomodulatory effects and inflammatory pathways. The mechanism (or mechanisms) of action of SVF when unraveled may help to shed further light on the pathophysiology of migraine and TTH.

The inflammatory pathways described in the pathogenesis of migraine may be addressed by many of the SVF constituents including M2 macrophages and ASCs. M2 macrophages are reported to have potent anti-inflammatory and immunoregulatory abilities, in particular, the production of key anti-inflammatory cytokines such as IL-10 and lipid mediators such as lipoxins. The inhibition of proinflammatory molecules by MSCs have also been well documented in number of diseases including acute lung injuries, myocardial infarction and cerebral ischemia [10]. A multitude of immunomodulatory effects of MSCs and ASCs have similarly been demonstrated in the literature by the regulation of indoleamine 2,3-dioxygenase, transforming growth factor -β1, human leukocyte antigen-G, prostaglandin E_2_ and tumor necrosis factor (TNF)-α-induced protein 6 [11]. Indeed MSCs have had a demonstrated neuroprotective effect on dopaminergic neurons, which may be hypersensitive in migraine, by a reduction in nitric oxide and TNF-α and messenger ribonucleic acid (RNA) expression of lipopolysaccharide-induced microglial activation, TNF-α and inducible nitric oxide synthase [12]. In general, it may be that the anti-inflammatory and immunoregulatory properties of the SVF are working to ‘mop up’ the IF that activates and sensitizes dural nociceptive neurons.

Although, as the pain of migraine does not eventuate and is long term this could suggest that the action of stromal cells are sufficient to correct deficiencies that predispose to migraine rather than merely being anti-inflammatory for the neurogenic inflammation. It could be presumed that the therapeutic efficacy of infused SVF relies on either or both the beneficial effects of locally engrafted SVF cells or systemic effects from secreted paracrine factors that diffuse into target tissues. The extravasations of systemically infused SVF and engraftment may also have occurred where they could exhibit both local trophic or paracrine activity in cell depleted areas.

SVF is also known to contain a high number of EPCs, in particular StroMed (unpublished data), which are reduced in both number and function in migraineurs sufferers. One proposed mechanism of recurrent migraine attacks is inflammation, hypoxia, shifts in vascular diameter and blood–brain barrier disruption injury of the vascular endothelium–and that this repeated injury is thought to exhaust the available supply of EPCs [13]. It has been suggested that endothelial dysfunction is causally related to migraine (rather than being a consequence of extensive triptan use or repeated migraine attacks), as endothelial dysfunction has been demonstrated in the systemic circulation of individuals with migraine of recent onset. It may be that the excess EPC in the SVF aid in the repair of this dysfunction. Similarly, stem cell depletion at the site of pathology has been demonstrated in progeria and may also be linked to other disorders [14].

Other potential mechanisms for the observed decrease in migraine frequency may be the ability of MSCs to modulate chronic pain. Guo *et al.* in 2011 [15] demonstrated that when neuropathic hyperalgesia was induced in rats and MSCs were injected both at the site and IV, they found they were able to produce an analgesic effect with both modalities although the local injection effect decreased by 4 weeks and the IV injection was maintained to the end of the study (4 months). The use of naloxone at the site initially reversed the analgesic effect, but was unable to at 4 months. In contrast, centrally acting naloxone at 4 months was able to reverse the analgesic effect demonstrating that MSCs may also act on a central mechanism to down regulate pain.

While we have observed a treatment that appears to lead to a relatively effective long-term reduction in migraines and TTH frequency in some patients, we will continue to monitor the patients to observe the length of time the patients will remain relatively migraine free before retreatment is necessary. These original case reports on the treatment of migraines and TTH by StroMed and SVF while encouraging requires further trials and proof of efficacy and understanding of the mechanism of action. This will require significant input from practitioners who are experts in the field.

## Consent

Written informed consent was obtained from the patients for publication of this case report and accompanying images. Copies of the written consents are available for review by the Editor-in-Chief of this journal.

## Competing interests

Matthew Bright and Shannon Hayne have no competing interests. The use of ultrasonic cavitation to obtain SVF from adipose is a filed Patent Co-operation (PCT) patent by Cell-Innovations. The use of SVF and stem cells for the use of migraines and TTH are filed PCT patents. Ralph Bright and Wayne Thomas are Directors of Cell-Innovations. Pelin Bright is a shareholder of Cell-Innovations.

## Authors’ contributions

MB, RB, PB and WT performed the procedures, collected the patient data, and assisted with completion of the manuscript. SH was a major contributor in writing the manuscript. All authors read and approved the final manuscript.
